# Docosahexaenoic acid enrichment in NAFLD is associated with improvements in hepatic metabolism and hepatic insulin sensitivity: a pilot study

**DOI:** 10.1038/ejcn.2017.9

**Published:** 2017-03-15

**Authors:** L Hodson, L Bhatia, E Scorletti, D E Smith, N C Jackson, F Shojaee-Moradie, M Umpleby, P C Calder, C D Byrne

**Affiliations:** 1 Oxford Centre for Diabetes, Endocrinology and Metabolism (OCDEM), Radcliffe Department of Medicine, University of Oxford, Oxford, UK; 2 Human Development and Health Academic Unit, Southampton, UK; 3 NIHR Southampton Biomedical Research Centre, University Hospital Southampton NHS Foundation Trust and University of Southampton, Southampton, UK; 4 Diabetes and Metabolic Medicine, Department of Nutritional Sciences, University of Surrey, Guildford, UK

## Abstract

**Background/Objective:**

Treatment of subjects with non-alcoholic fatty liver disease (NAFLD) with omega-3 polyunsaturated fatty acids (FAs) suggests high levels of docosahexaenoic acid (DHA) tissue enrichment decrease liver fat content. We assessed whether changes in erythrocyte DHA enrichment (as a surrogate marker of changes in tissue enrichment) were associated with alterations in hepatic *de novo* lipogenesis (DNL), postprandial FA partitioning and hepatic and peripheral insulin sensitivity in a sub-study of the WELCOME trial (Wessex Evaluation of fatty Liver and Cardiovascular markers in NAFLD (non-alcoholic fatty liver disease) with OMacor thErapy).

**Subjects/Methods:**

Sixteen participants were randomised to 4 g/day EPA+DHA (*n*=8) or placebo (*n*=8) for 15–18 months and underwent pre- and post-intervention measurements. Fasting and postprandial hepatic FA metabolism was assessed using metabolic substrates labelled with stable-isotope tracers (^2^H_2_O and [U^13^C]palmitate). Insulin sensitivity was measured by a stepped hyperinsulinaemic-euglycaemic clamp using deuterated glucose. Participants were stratified according to change in DHA erythrocyte enrichment (< or ⩾2% post intervention).

**Results:**

Nine participants were stratified to DHA⩾2% (eight randomised to EPA+DHA and one to placebo) and seven to the DHA<2% group (all placebo). Compared with individuals with erythrocyte <2% change in DHA abundance, those with ⩾2% enrichment had significant improvements in hepatic insulin sensitivity, reduced fasting and postprandial plasma triglyceride concentrations, decreased fasting hepatic DNL, as well as greater appearance of ^13^C from dietary fat into plasma 3-hydroxybutyrate (all *P*<0.05).

**Conclusions:**

The findings from our pilot study indicate that individuals who achieved a change in erythrocyte DHA enrichment ⩾2% show favourable changes in hepatic FA metabolism and insulin sensitivity, which may contribute to decreasing hepatic fat content.

## INTRODUCTION

Non-alcoholic fatty liver disease (NAFLD) is a spectrum of liver fat-related conditions that increase risk of chronic metabolic disease such as type 2 diabetes and cardiovascular disease, with obesity and insulin resistance (IR) being well-documented risk factors.^1^ IR appears to be a key mediator in the initiation and progression of NAFLD, mainly through adverse changes in glucose, fatty acid (FA) and lipoprotein metabolism;^2^ liver fat content appears to be the best independent predictor of peripheral and hepatic IR.^3^ A net retention of triglyceride (TG) within the liver is a prerequisite for the development of NAFLD and increased FA flux to the liver, increased FA synthesis within the liver, and decreased hepatic FA oxidation have all been implicated in NAFLD development.^1, 4^

High-dose omega-3 FA (eicosapentaenoic acid (EPA) and docosahexaenoic acid (DHA)) are a licensed treatment to reduce plasma TG concentrations.^5^ As NAFLD is associated with an overproduction of very low-density lipoprotein (VLDL)-TG,^6^ it is plausible that one contributor to the hypotriglyceridemic effect of omega-3 FA is through a lowering of liver fat content. We^7^ and others^8, 9^ have reported that omega-3 FA have the potential to decrease liver fat. In addition to potentially lowering liver fat, short-term omega-3 FA treatment has been reported to improve whole-body insulin sensitivity.^8, 9^ However, the effects of omega-3 FA on insulin sensitivity in the context of NAFLD remains unclear, as some studies report neutral or negative results,^10, 11^ despite a concomitant reduction in NAFLD severity.^12^

The current study is a pre-specified sub-study of the WELCOME* randomised double-blind, placebo-controlled trial.^7, 13^ The aim of this pilot sub-study was to test if a pre-specified increase (⩾2%) in erythrocyte enrichment of DHA^7, 13^ was associated with changes in hepatic FA synthesis, postprandial FA partitioning and hepatic and peripheral insulin sensitivity.

## Materials and methods

**Figure 1:**
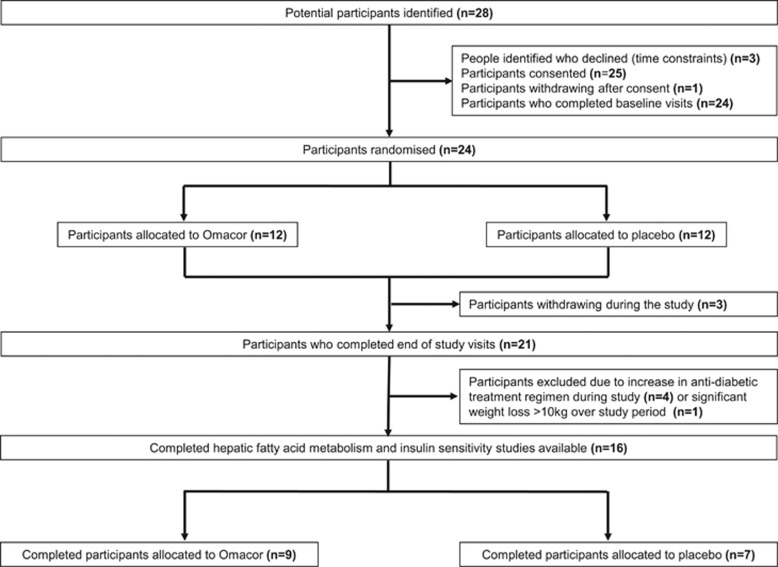
Consort diagram showing recruitment for the WELCOME sub-study and the numbers of subjects available for hepatic fatty acid metabolism and insulin sensitivity studies. See text for the reasons for withdrawal from the study.

Twenty-four individuals recruited from the main trial were randomly allocated to the sub-study (*n*=12 randomised to EPA+DHA, 4 g/day and *n*=12 randomised to placebo (olive oil, 4 g/day; see Figure 1).^7, 13^ The duration of intervention was 15–18 months and inclusion and exclusion criteria were described previously.^13^ For the main trial patients were randomised according to standardised procedures (computerised block randomisation in blocks of four, either to trial medication or placebo were used) by a research pharmacist at University Hospital Southampton NHS Foundation Trust.^13^ This randomisation strategy was maintained for the sub-study. The study was approved by the Southampton and South West Hampshire Local Research Ethics Committee (REC 08/H0502/165). All subjects gave written informed consent for both the main trial and the sub-study.

Three participants withdrew from the sub-study before completing all tests. Four patients with diabetes were not included in the analysis as their anti-diabetic regimens increased between baseline and end-of-study tests, which would have influenced change in insulin sensitivity measurements. Similarly, one participant who lost >10 kg in weight over the course of the trial was also excluded.

We compared participants who showed an increase in erythrocyte DHA enrichment of 2% or greater (DHA⩾2%) between baseline and end of the study with participants showing little change in erythrocyte DHA enrichment (DHA<2%). In the DHA⩾2% group eight participants had been randomised to EPA+DHA intervention and one participant had been randomised to placebo; the latter had a 4.2% increase in erythrocyte DHA between baseline and end of the study. All seven subjects in the DHA<2% group had been randomised to the placebo.

### Experimental procedures

Blood samples were taken after an overnight fast (12 h) and serum separated within 1 h to undergo routine biochemical assay.^7^ Blood pressure was measured using a Marquette Dash 3000 monitor (GE Healthcare, Bucks, UK), body composition by dual-energy X-ray absorptiometry and liver fat content measured at baseline and end of the study by magnetic resonance spectroscopy.^13^

### Red blood cell FA composition

To determine specific FA composition erythrocyte ghosts were prepared, membranes isolated, total lipids isolated, FA methyl esters prepared and FA compositions determined by gas chromatography (GC), as described elsewhere.^14^

### Assessment of whole-body and hepatic insulin sensitivity

Insulin sensitivity was measured using a two-step hyperinsulinaemic-euglycaemic clamp with a deuterated glucose (6,6 ^2^H_2_ glucose) infusion^15, 16^ to assess hepatic and peripheral insulin sensitivity. Briefly, subjects arrived at the NIHR Wellcome Trust Clinical Research Facility after an overnight (12 h) fast and intravenous cannulae were placed in both antecubital veins for sampling and infusion of labelled glucose and insulin during the two-step hyperinsulinaemic-euglycaemic clamp.^15, 16^

Glucose isotopic enrichment was determined from deproteinised plasma using the methoxime-trimethlysilyl ether derivative, which was measured by GC mass spectrometry using selected ion monitoring.^15^ A modified version of the equations formulated by Steele *et al.*^17^ for non-steady state was used to calculate total rate of appearance (Ra) of glucose, endogenous glucose production (at basal and low-dose insulin stage) and rate of disappearance (Rd) as well as metabolic clearance rate (at the high-dose insulin stage) of glucose adjusted to fat-free mass μmol/kg/min. For the Steele equations, 65% was used as the effective fraction and 0.22 l/kg as the distribution volume of glucose to calculate Ra and Rd.^17, 18^ Plasma glucose and tracer to tracee ratio time courses were smoothed using optimal segment smoothing method.^19^ We also measured a validated index of hepatic insulin sensitivity by dividing the basal endogenous glucose production rates by the fasting insulin concentration.^20^

### Fasting hepatic DNL and postprandial hepatic FA partitioning

Approximately 2 weeks after the assessment of whole-body and hepatic insulin sensitivity, subjects underwent a postprandial study day as previously described.^21^ Briefly, to assess fasting hepatic *de novo* lipogenesis (DNL), subjects consumed deuterated water (^2^H_2_O; 3 g/kg body water) the evening before the study day to achieve a body water enrichment of 0.3%.^21^ Subjects arrived at the Clinical Research Facility after an overnight fast and were fed a standard test meal that contained 200 mg of [U^13^C]palmitic acid (isotope purity 97%, CK Gas Products Ltd, Hook, UK) to trace the fate of dietary FA.^21^ Serial blood and breath samples were taken throughout the 5 h postprandial period.

### Biochemical analysis

Whole blood was collected into heparinized syringes (Starstedt, Leicester, UK) and plasma was rapidly separated for the measurement of metabolite and insulin concentrations.^21^ Separations of chylomicrons of Svedberg floatation rate (S_f_) >400 and VLDL-rich fraction (S_f_ 20–400) were made by sequential flotation using density gradient ultracentrifugation and the S_f_ 20–400 fraction was then further separated by immunoaffinity chromatography.^22, 23^

### FA analysis and isotopic enrichment

To determine specific FA composition and isotopic enrichment, total lipids were extracted from plasma and lipoproteins and FA methyl esters were prepared.^22^ FA compositions in these fractions were determined by GC, and palmitate concentrations were calculated as described elsewhere.^21^

^13^C/^12^C ratios in plasma non-esterified fatty acid, S_f_ >400 (chylomicron-TG), S_f_ 20–400-TG (TRL) and VLDL-TG FA methyl ester derivatives were determined using a ‘Delta Plus XP’ GC-combustion-isotope ratio MS (GC-C-IRMS) (Thermo Electron Corporation, Bremen, Germany) and ^13^C concentrations calculated as previously described.^23^

To assess ketone body production arising from the oxidation of dietary [U^13^C]palmitate, we measured isotopic enrichment of [^13^C] in plasma 3OHB.^21^ Fasting hepatic DNL was assessed based on the incorporation of deuterium from ^2^H_2_O in plasma water (Finnigan GasBench-II, ThermoFisher Scientific, Hemel Hempstead, UK) into VLDL-TG palmitate using GC mass spectrometry.^21^ The concentration of VLDL-TG derived from DNL was determined by multiplying %DNL and the concentration of TG in VLDL.^24^

### Sample size, calculations and statistical analysis

The sample size for the main study^7^ was powered to detect a change in liver fat content, as described previously;^13^ the sub-study reported here was run as a hypothesis-generating pilot study. Areas under the curve were calculated by the trapezoid method and were divided by the relevant period to give a time-averaged value. Data were analysed using SPSS for Windows v20 (SPSS, Chertsey, UK). Statistical significance was set at *P*<0.05. Data are reported as mean (s.e.m.) unless otherwise stated. All data sets were tested for normality according to the Shapiro–Wilk test. Baseline results were compared with end of study results using a paired *t*-test or the Wilcoxon signed rank test for non-parametric data. Comparisons between the two groups were undertaken using independent *t*-test or Mann–Whitney *U-*test for non-parametric data.

## Results

### Subject characteristics

Baseline and end of study characteristics of participants with a change in erythrocyte DHA enrichment of ⩾2% (*n*=9) or <2% (*n*=7) are shown in Table 1. One participant randomised to the placebo group had a significant increase in erythrocyte EPA and DHA enrichment (0.8 and 4.2%, respectively) between baseline and end of study measurements; the increase is most likely because they started consuming more oily fish or over-the-counter fish oil capsules during the course of the trial. There was no significant change in body mass index or body fat percentage between baseline and end of study in either group (Table 1). Baseline liver fat content ranged from 5.3 to 85% and although liver fat decreased to a greater extent in the DHA⩾2% compared with the DHA<2% group, this difference did not reach statistical significance.

We measured erythrocyte FA composition as a surrogate marker for tissue, specifically liver, FA enrichment.^25, 26^ The DHA⩾2% group had a significant (*P*<0.001) change in the erythrocyte enrichment of EPA (by >300%) and DHA (by 92%) between baseline and end of study measurements; there was no change in the DHA<2% group (Table 1).

There was no difference between baseline and end of study in the fasting plasma concentrations of glucose, insulin, non-esterified fatty acid, total, low-density lipoprotein cholesterol or high-density lipoprotein cholesterol (Table 2). Fasting plasma TG and VLDL-TG concentrations were significantly (*P*<0.001) decreased by 0.6 mmol/ l and 0.7 mmol/l, respectively, in the DHA⩾2% group, while concentrations remained unchanged in the DHA<2% group (Table 2).

We measured the incorporation of newly synthesised palmitate (DNL) into VLDL-TG in the fasting state and found no difference in the relative contribution between baseline and end of study in either group (Table 2). However, when expressed as the absolute concentration of VLDL-TG derived from DNL there was a significant (*P*<0.05) decrease in the DHA⩾2% group between baseline and end of study measurements but there was no change in the DHA<2% group (Table 2).

In line with the fasting data, there was a significant (*P*<0.001) decrease in postprandial plasma TG and VLDL-TG concentrations between baseline and end of study measurements in the DHA⩾2% group but not the DHA<2% group (Table 2). There was a significant (*P*<0.05) decrease in plasma 3OHB concentration over the postprandial period in the DHA⩾2% group (Table 2).

We included [U^13^C]palmitate as part of the mixed test meal, to trace the fate of dietary FA over the 5 h postprandial period. We observed a significant (*P*<0.05) decrease in the appearance of [U^13^C]palmitate in the plasma non-esterified fatty acid pool in the DHA<2% group, but not in the DHA⩾2% group (Table 2). There was a significant (*P*<0.01) decrease in the appearance of [U^13^C]palmitate in VLDL-TG and a significant (*P*<0.01) increase in the incorporation of ^13^C into plasma 3OHB in the DHA⩾2% group whilst the DHA<2% group remained unchanged (Table 2).

Whole-body insulin sensitivity (M-value) and peripheral glucose disposal (Rd) during the high-dose insulin stage did not change in either the DHA⩾2% or DHA<2% group between baseline and end of study (Table 3). Hepatic insulin sensitivity significantly (*P*<0.01) increased in the DHA⩾2% group over the course of the study, with no change being observed in the DHA<2% group (Table 3). We investigated whether a change in liver fat percentage was associated with changes in insulin sensitivity. In exploratory analyses, we stratified the cohort into two equal groups by the median change in liver fat percentage during the trial. Group 1 represented a ‘high’ reduction in liver fat (range −3% to −53%), whilst Group 2 represented minimal change or increase in liver fat (range −1.3% to +33%). When we analysed the difference in percentage suppression of endogenous glucose production at the low-dose insulin step between the groups (as another measure of hepatic insulin sensitivity), percentage suppression was significantly better in Group 1 vs Group 2 (13.7 vs −3.8% (95% confidence interval 1.4, 33.5, *P*<0.05)). However, the difference in percentage increase of glucose disposal (measure of whole-body insulin sensitivity) was not significantly different (Group 1, 26.7% vs Group 2, 8.7% (95% confidence interval −56.2, 92.3, P=0.61)).

**Figure 2:**
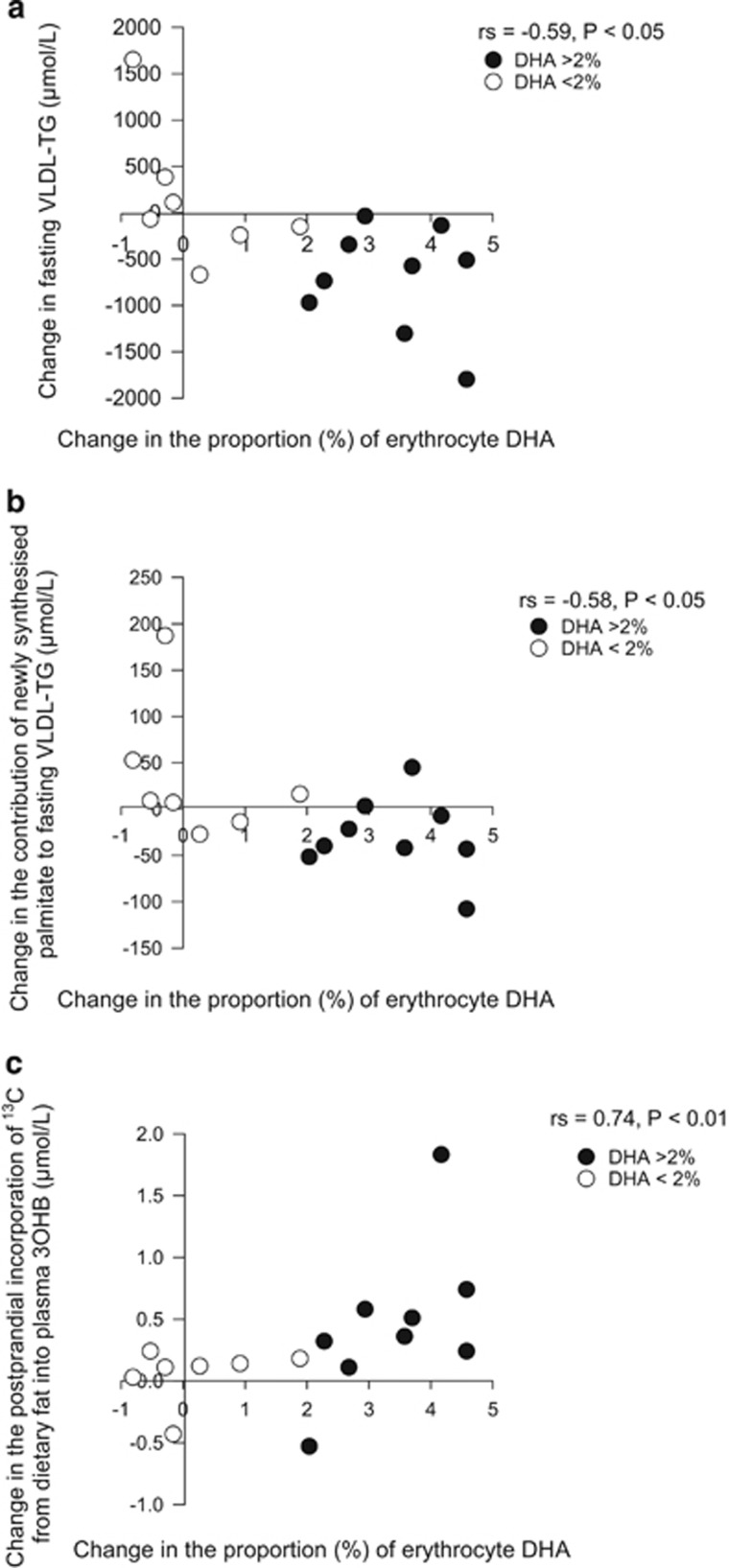
Correlations between change in erythrocyte DHA (%) and change in fasting plasma VLDL-triacylglycerol (TG) concentrations (μmol/l) (**a**); change in erythrocyte DHA (%) and change in the fasting contribution (μmol/l) of DNL fatty acids to VLDL-TG (**b**); and change in erythrocyte DHA (%) and change in postprandial plasma [^13^C]3OHB concentrations (μmol/L) (**c**).

We found an inverse association between change in erythrocyte DHA enrichment and change in fasting plasma VLDL-TG concentrations (r_s_=−0.59, *P*<0.05; Figure 2a). We also assessed the association between change in hepatic DNL and change in erythrocyte DHA enrichment and found an inverse association (r_s_=−0.58, *P*<0.05; Figure 2b). Lastly, we assessed the association between change in erythrocyte DHA status and the change in the incorporation of ^13^C from dietary fat into plasma 3OHB and found a significant positive correlation (*r*_s_=0.74, *P*<0.01; Figure 2c).

## Discussion

We report here data demonstrating that individuals with NAFLD, who have an increase in erythrocyte DHA enrichment of ⩾2% (as a marker of tissue enrichment^25, 26^) through treatment with omega-3 FA, show favourable changes in both hepatic insulin sensitivity and hepatic FA metabolism. Although erythrocyte DHA enrichment ⩾2% was associated with a nonsignificant (26%) decrease in liver fat content, hepatic DNL significantly decreased while hepatic FA oxidation and hepatic insulin sensitivity significantly increased. Further analysis of our data revealed a reduction in liver fat was also significantly associated with improved hepatic, but not peripheral, insulin sensitivity. Given that increased liver fat is associated with defects in insulin-mediated suppression of glucose production,^27^ our study extends this observation by showing a significant improvement in hepatic insulin sensitivity over 18 months in association with a reduction in liver fat.

Liver fat content is associated with an overproduction of VLDL, which may result in dyslipidemia^6^ and a decrease in liver fat content may contribute to a decrease in VLDL-TG production. In line with previous work,^28, 29^ we found a significant decrease in fasting and postprandial VLDL-TG concentrations in the DHA⩾2% group only. Evidence from kinetic studies has demonstrated that omega-3 FA decrease TRL particle concentrations by decreasing hepatic TRL apoB-100 synthesis and secretion rates and increasing TRL-to-low-density lipoprotein conversion.^28, 29^ We did not measure TRL production, secretion or conversion rates in the current study.

FA availability is a determinant of VLDL-TG production. We found the absolute contribution of FA derived from hepatic DNL to VLDL-TG in the fasting state was significantly decreased in the DHA⩾2% group. A plausible explanation for the decrease in DNL is that omega-3 FA inhibit sterol regulatory element-binding protein 1c (SREBP1c), which leads to a downregulation in the expression of several key lipogenic genes involved in FA synthesis.^30^ We observed a significant decrease in the appearance of [^13^C]palmitate in VLDL-TG over the postprandial period in the DHA⩾2% group, with no change in the DHA<2% group, suggesting a change in the intrahepatic partitioning of dietary FA. In support of this observation, we found a notable increase in the appearance of ^13^C (from dietary FA) into plasma 3OHB, suggesting intrahepatic metabolism was being moved away from esterification toward ketogenic pathways over the postprandial period. These observations are also in line with the liver becoming more insulin sensitive in the DHA⩾2% group.

Supplementation with EPA+DHA has been reported to decrease liver fat in some^7, 8^ but not all^31^ studies. In the present study, individuals who increased their erythrocyte DHA enrichment by ⩾2% had, on average a nonsignificant 26% decrease in liver fat content. In the WELCOME Study results^7, 32^ we noted some individuals benefitted markedly from treatment with 4 g/day DHA+EPA, while others derived no benefit; a result which cannot be explained through lack of adherence to DHA+EPA treatment.

It is important to consider the strengths and limitations of our study. Although the sample size is small in this proof of concept pilot study, we have undertaken a randomised double-blind, placebo-controlled trial testing the effects of the high-dose omega-3 FA intervention over a minimum period of 15 months. Subjects consumed either a combination of EPA+DHA or placebo (olive oil). Recent work by Allaire *et al.*^33^ reported, in a head-to-head comparison of EPA and DHA, that the latter was more effective in modulating markers of inflammation and blood lipids. It would be of interest to study the effect of a DHA treatment only on hepatic metabolism and insulin sensitivity given improvements in hepatic insulin sensitivity, and hepatic FA partitioning were noted in individuals with a ⩾2% change in erythrocyte DHA abundance. Although *in vitro* cellular work has suggested oleic acid, when compared with palmitic acid has a protective role against insulin resistance^34^ it is likely subjects in the placebo group of the current study were not supplemented with high enough doses of oleic acid to have an effect. Subjects in the placebo group consumed 4 g olive oil per day, which provided 2.4 g oleic acid per day in addition to their habitual diet; oleic acid is very prevalent in many foods of both plant and animal origin. Subjects underwent very extensive phenotyping to characterise key aspects of liver metabolism and gold-standard techniques were applied to assess liver fat, insulin sensitivity and hepatic FA partitioning. As our postprandial period was only of 5 h duration, it is plausible that we have underestimated the effects, and had we traced the meal for longer, or investigated hepatic FA partitioning after multiple meals, we may have found greater divergence between the groups. We also only measured hepatic DNL in the fasting state and it would be of interest to measure hepatic DNL in the postprandial state. Additionally, we cannot determine how early or in what order changes in liver fat, hepatic FA synthesis and partitioning, and hepatic insulin sensitivity occurred.

In conclusion, individuals with NAFLD who increase erythrocyte DHA enrichment (⩾2% as a marker for tissue enrichment) show notable changes in hepatic insulin sensitivity and hepatic FA metabolism that favour decreased hepatic DNL and increased hepatic FA oxidation which would be expected to decrease hepatic TG synthesis and storage. Variable tissue enrichment with DHA may in part, explain the differential effects of omega-3 FA treatment on hepatic fat quantity via the different pathways influenced by omega-3 FA treatment. Subjects achieving higher DHA enrichment ⩾2% tended to have higher hepatic insulin sensitivity, lower fasting hepatic DNL and higher levels of hepatic fat oxidation.

## Figures and Tables

**Table 1:** Comparison between baseline and end of study participant characteristics in non-diabetic participants grouped by change in erythrocyte DHA enrichment (⩾2% or <2%)

*Variables*	*DHA ⩾2% (*n=*9)*		*DHA <2% (*n=*7)*	
	*Baseline*	*End*	*Baseline*	*End*
Group (treatment/placebo)	8/1		0/7	
Sex (M/F)	5/4		6/1	
Age (year)	45.7±4.4		56.7±2.5	
Weight (kg)	94.9±5.4	95.4±5.6	98.3±1.6	95.9±2.6
BMI (kg/m^2^)	33.3±1.2	33.4±0.9	32.8±1.3	31.8±0.8
Waist circumference (cm)	110.8±2.9	110.9±2.9	111.8±1.6	109.6±1.2
DEXA % body fat	40.0±2.1	39.8±2.2	33.8±3.3	34.8±2.6
MRS liver fat (%)	34.4±8.5	25.3±6.1	18.9±5.4	15.9±12.3
MRI visceral mass (kg)	3.36±0.43	3.53±0.32	3.79±0.34	3.41±0.19
MAP (mmHg)	102.7±3.6	99.4±3.4	104.2±4.8	102.9±4.2
HbA1c (% total Hb)	5.8±0.1	5.8±0.2	6.0±0.2	5.9±0.3
Erythrocyte EPA (%)	0.82±0.13	3.44±0.47^a^	1.00±0.10	0.90±0.08
Erythrocyte DHA (%)	3.68±0.60	7.08±0.47^a^	4.62±0.40	4.81±0.26

Abbreviations: Abbreviations: BMI, body mass index; DEXA, dual-energy X-ray absorptiometry; DHA, docosahexaenoic acid; End, end of study; EPA, eicosapentaenoic acid; HbA1c, glycated haemoglobin A1c; MAP, mean arterial pressure; MRI, magnetic resonance imaging; MRS, magnetic resonance spectroscopy.

Data presented as mean±s.e.m.

a *P*<0.001 between baseline and end of study measurements within the respective groups.

**Table 2:** Comparison between baseline and end of study blood metabolites and markers of hepatic fatty acid synthesis and partitioning in non-diabetic participants stratified by change in erythrocyte DHA enrichment (⩾2% or <2%)

	*DHA ⩾2% (*n=*9)*		*DHA <2% (*n=*7)*	
	*Baseline*	*End*	*Baseline*	*End*
*Fasting plasma concentrations*				
Glucose (mmoll^−1^)	5.6±0.2	5.9±0.3	5.6±0.2	5.5±0.2
Insulin (mUl^−1^)	35±6	33±8	40±11	21±3
Total cholesterol (mmoll^−1^)	4.5±0.3	4.7±0.4	5.1±0.6	4.6±0.3
HDL-cholesterol (mmoll^−1^)	1.2 (0.7, 1.4)	1.3 (0.9, 1.4)	1.0 (0.9, 1.2)	1.1 (1.0, 1.1)
LDL-cholesterol (mmoll^−1^)	2.6±0.2	3.0±0.3	2.5±0.3	2.5±0.4
TG (mmoll^−1^)	2.1±0.3	1.5 ±0.2^a^	2.3±0.4	2.6±0.5
VLDL-TG (μmoll^−1^)	1348±212	635±88^a^	1497±319	1641±429
NEFA (μmoll^−1^)	550±55	570±74	653±79	663±80
3OHB (μmoll^−1^)	83±13	58±15	93±19	106±34
				
*Fasting contribution of hepatic DNL to VLDL-TG*				
Hepatic DNL (%)	14.8±3.6	15.8±2.2	11.1±1.9	16.1±2.4
VLDL-TG derived from DNL (μmoll^−1^)	61.0±14	31.0±6.0^b^	51.2±14.5	84.1±30.5
				
*Time-averaged postprandial plasma concentrations*				
Glucose (mmoll^−1^)	6.2±0.4	6.5±0.4	6.1±0.4	6.1±0.2
Insulin (mUl^−1^)	94±15	88±19	107±33	67±11
TG (mmoll^−1^)	2.4±0.3	1.8±0.2^c^	2.7±0.4	2.9±0.6
NEFA (μmoll^−1^)	373±47	368±50	389±55	382±42
3OHB (μmoll^−1^)	68±10	46±8^b^	76±17	69±18
Chylomicron-TG (μmoll^−1^)	180±49	149±37	268±121	350±158
VLDL-TG (mmoll^−1^)	1.5±0.2	0.8±0.1^c^	1.9±0.3	1.7±0.4
				
*Time-averaged postprandial plasma and breath* ^*13*^*C concentrations*				
[^13^C]Chylomicron-TG (μmoll^−1^)	0.6±0.2	0.4±0.1	1.0±0.6	1.0±0.6
[^13^C]NEFA (μmoll^−1^)	0.22±0.09	0.20±0.08	0.16±0.05	0.07±0.02^b^
[^13^C]VLDL-TG (μmoll^−1^)	0.6±0.1	0.3±0.1^b^	0.6±0.2	0.7±0.2
[^13^C]3OHB (μmoll^−1^)	0.1±0.1	0.6±0.2^b^	0.2±0.1	0.2±0.1

Abbreviations: 3OHB, 3-hydroxybutyrate; DHA, docosahexaenoic acid; DNL, *de novo* lipogenesis; End, end of study; FFM, fat-free mass; HDL, high-density lipoprotein; LDL, low-density lipoprotein; NEFA, non-esterified fatty acid; TG, triglyceride; VLDL, very low-density lipoprotein.

Data presented as mean±s.e.m. or median (25th, 75th percentiles).

a *P*<0.01;

b *P*<0.05; and

c *P*<0.001 between baseline and end of study measurements within the respective groups.

**Table 3:** Comparison between baseline and end of study markers of hepatic and whole-body insulin sensitivity in non-diabetic participants grouped by change in erythrocyte DHA enrichment (⩾2% or <2%)

*Variables*	*DHA ⩾2% (*n=*9)*		*DHA <2% (*n=*7)*	
	*Baseline*	*End*	*Baseline*	*End*
Basal endogenous glucose production (Ra; μmol/min/kg FFM)	15.2±0.8	14.4±0.7	13.4±0.7	14.0±1.0
Low-dose insulin EGP (μmol/min/kg FFM)	8.7±0.9	7.8±0.7	7.1±0.5	6.7±1.0
High-dose insulin total body glucose disposal (Rd; μmol/min/kg FFM)	35.0±3.1	34.3±4.2	30.4±3.5	35.9±5.5
High-dose insulin total body glucose clearance (MCR; ml/min/kg FFM)	7.17±0.84	6.79±0.75	6.12±0.73	7.26±1.16
M-value (ml/min/kg)	3.22±0.33	3.21±0.34	3.23±0.61	3.77±0.73
Hepatic insulin sensitivity index (μmol/min/kg FFM; mU/l) × 10^2^	0.54 (0.36, 0.82)	0.63 (0.42, 0.89)^a^	0.52 (0.30, 0.67)	0.55 (0.46, 1.42)
Adipose-IR × 10^-2^	75.5±11.0	109.0±38.9	110.0±27.6	67.9±10.1

Abbreviations: DHA, docosahexaenoic acid; EGP, endogenous glucose production; FFM, fat-free mass; IR, insulin resistance; M-value, glucose infusion rate; MCR, metabolic clearance rate; Ra, Rate of appearance of glucose; Rd, rate of glucose disposal.

Data presented as mean±s.e.m. or median (25th, 75th percentiles).

a *P*<0.01 between baseline and end of study measurements within the respective groups.
