# Erratum: Docosahexaenoic acid enrichment in NAFLD is associated with improvements in hepatic metabolism and hepatic insulin sensitivity: a pilot study

**DOI:** 10.1038/ejcn.2017.145

**Published:** 2017-08-16

**Authors:** L Hodson, L Bhatia, E Scorletti, D E Smith, N C Jackson, F Shojaee-Moradie, M Umpleby, P C Calder, C D Byrne

**Keywords:** Fat metabolism, Clinical trials, Non-alcoholic fatty liver disease, Nutritional supplements

**Correction to:** *European Journal of Clinical Nutrition* (2017) **71**, 973–979; doi:10.1038/ejcn.2017.9; published online 15 March 2017

Updated online 16 August 2017: This article was originally published under a standard *EJCN* license, but has now been made available under a CC BY 4.0 license. The PDF and HTML versions of the paper have been modified accordingly.

